# Auto-aggressive metallic mercury injection around the knee joint: a case report

**DOI:** 10.1186/1471-2482-11-31

**Published:** 2011-11-17

**Authors:** Joerg Friesenbichler, Werner Maurer-Ertl, Patrick Sadoghi, Elisabeth Wolf, Andreas Leithner

**Affiliations:** 1 Department of Orthopaedic Surgery, Medical University of Graz, Auenbruggerplatz 5, 8036 Graz, Austria

## Abstract

**Background:**

Accidental or intentional subcutaneous and/or intramuscular injection of metallic mercury is an uncommon form of poisoning. Although it does not carry the same risk as mercury vapour inhalation, it may cause destructive early and late reactions.

**Case Presentation:**

Herein we present the case of a 29-year-old male patient who developed an obsessive-compulsive disorder causing auto-aggressive behaviour with injection of elemental mercury and several other foreign bodies into the soft tissues around the left knee about 15 years before initial presentation. For clinical examination X-rays and a CT-scan of the affected area were performed. Furthermore, blood was taken to determine the mercury concentration in the blood, which showed a concentration 17-fold higher than recommended. As a consequence, the mercury depots and several foreign bodies were resected marginally.

**Conclusion:**

Blood levels of mercury will decrease rapidly following surgery, especially in combination with chelating therapy. In case of subcutaneous and intramuscular injection of metallic mercury we recommend marginal or wide excision of all contaminated tissue to prevent migration of mercury and chronic inflammation. Nevertheless, prolonged clinical and biochemical monitoring should be performed for several years to screen for chronic intoxication.

## Background

There are several reports in the literature concerning mercury intoxication with Wolfgang Amadeus Mozart as the most prominent possible victim [1]. In most cases, mercury is predominantly absorbed by the respiratory system, particularly by inhalation of the vapour, which is produced at room temperature [2-6]. Following inhalation, mercury damages the lung tissue. Furthermore, a large part of mercury is absorbed by the pulmonary vasculature and distributed to further organs. Mercury also passes the blood-brain barrier and remains in the nervous system before being oxidized [6-8].

Subcutaneous and intramuscular injection of elemental mercury is of rare occurrence, although in most cases it may be injected voluntarily in the setting of attempted suicide or in context of psychiatric diseases [4,5,7,9-16]. Prasad et al. [10] reported the subcutaneous injection of mercury for ethnic reasons and Lech and Goszcz [3] related a case of mercury intoxication due to ingestion with suicidal intent.

After entering the body following intravenous or even intra-arterial injection elemental mercury is transported to the liver, the spleen, the kidneys and the brain [2,4,6,7,9,12,16]. At these locations, mercury is oxidized into divalent mercury (mercurous and mercuric), which is able to cohere with thiol-groups of enzyme systems, causing tissue damages [6,7,9,16].

Herein we present the case of a 29-year-old male patient who developed an obsessive-compulsive disorder (OCD) causing auto-aggressive behaviour with injection of elemental mercury and other foreign bodies into the left upper and lower leg, 15 years before initial presentation.

## Case Presentation

In October 2010, a 20-year-old patient was referred to our clinic complaining about stinging pain in the left thigh and the knee. One year before, he was treated due to an extra-skeletal osteosarcoma of the right thigh with a wide resection for local tumour control and poly-chemotherapy (EURAMOS scheme) and local radiation therapy.

Currently, the patient reported about the development of an obsessive-compulsive disorder (OCD) 15 years before, causing auto-aggressive behaviour. In the context of this disease, the patient injected elemental metallic mercury from a clinical thermometer into the soft tissues around the left knee and the upper and lower leg. Furthermore, he pricked 2 spikes and approximately 17 spicules into his thigh during a period of several months and left them in situ. Therefore, an infection of the soft tissues developed, which was treated surgically at an external hospital. Furthermore, a therapy with antidepressant agents was initiated.

**Figure 1:**
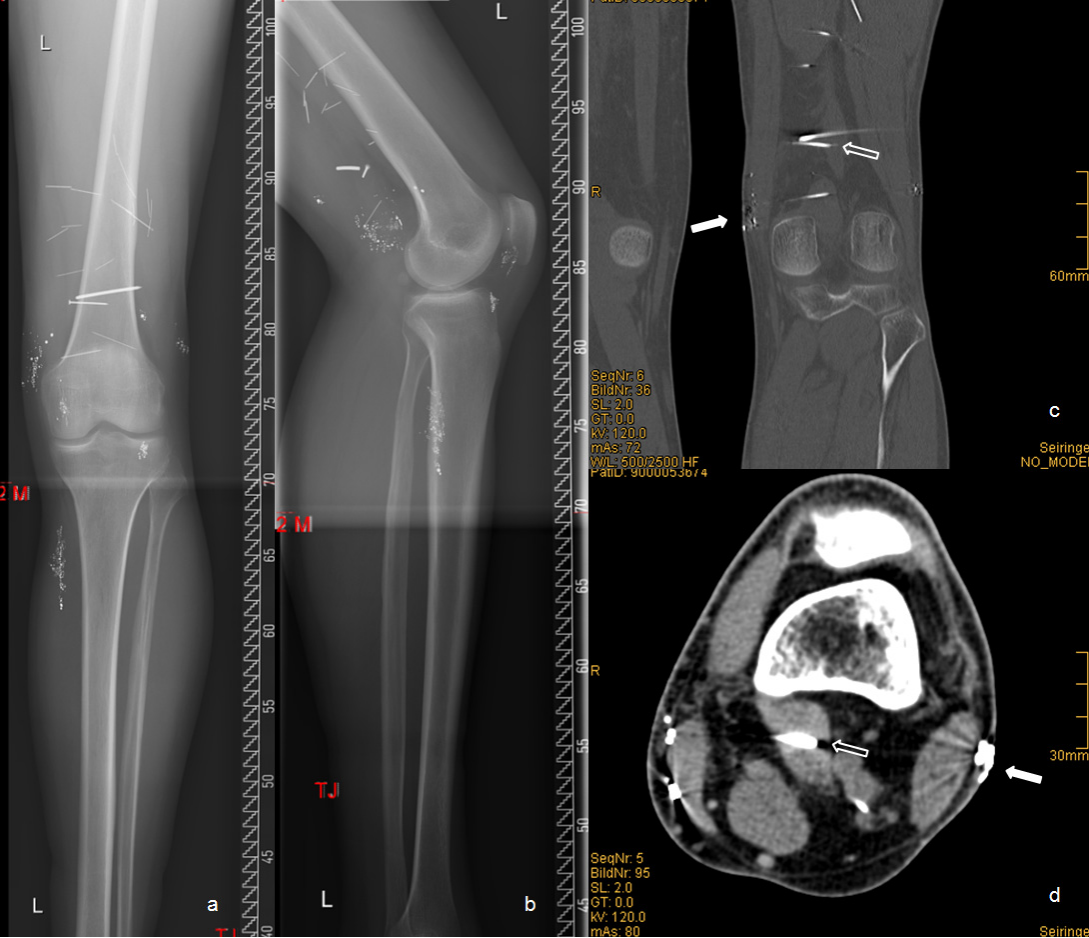
**a-d. a&b) Preoperative anteroposterior and lateral radiographs of the left femur and tibia showing multiple radiopaque particles, spikes and spicules in the soft tissues around the knee**. c&d) Coronal and transversal CT scan of the left knee and the affected soft tissues showing several metallic foreign bodies (hollow arrow: 2 spikes) and mercury depots (white arrow).

Nevertheless, plain radiographs and computed tomography (CT) of the affected area showed significant volumes of radiopaque foreign material (depots of mercury) and several foreign bodies (2 spikes and 17 spicules; Figure 1a-d) in the soft tissues. The staging investigations performed for the osteosarcoma (local X-rays of the right thigh, CT scan of thorax, abdomen and pelvis) which are performed regularly never showed mercury depots elsewhere in the body.

**Figure 2:**
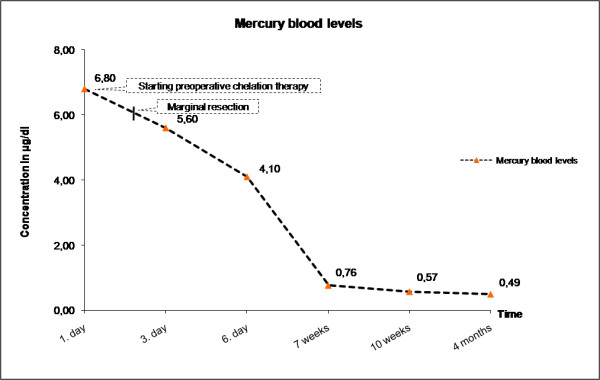
**Diagram showing the blood levels of mercury before and after excision of the mercury deposits, as well as under chelating therapy using DMPS**. Orange arrow: time of surgery.

Blood was taken to determine mercury concentration, the C-reactive protein (CRP) and renal function parameters preoperatively. While the renal parameters revealed no pathologic findings, the blood level of mercury (6,80 μg/dl) was 17-fold higher than recommended by the reference laboratory (standard: 0-0,40 μg/dl; Figure 2). The CRP was increased, additionally. Surprisingly, there were no neurological symptoms or other signs of systemic poisoning, which are known to occur following long time exposure to elemental mercury. Moreover, no context could be found between metallic mercury injection and the extra-skeletal osteosarcoma, neither in the current case, nor in the literature.

As a consequence, foreign bodies (2 spikes and 4 spicules) and several mercury depots were resected marginally, except of 13 spicules, which had to be left in situ due to the diffuse distribution. Excision of these foreign bodies would have caused too much soft tissue damage of the extensor mechanism. Furthermore, a therapy with a chelating agent (2,3-dimercaptopropane-1-sulfonate, DMPS) was initiated for 2 months and the therapy with antidepressant agents was continued.

Haematoxylin and Eosin (H&E) stained sections of the specimen showed cicatricial tissue, granulomas caused by foreign bodies, multiple abscesses and black amorphous material (mercury depots) surrounded by the foreign-body reaction.

At 6-months follow-up, the patient reported less pain in the left upper leg, the blood concentrations of mercury and CRP had decreased significantly (0,49 μg/dl at 4 months of follow-up) (Figure 2). The patient did not report any adverse reactions to the chelation agent and he was able to ambulate without any facilities. Clinical and biochemical observation was continued.

## Conclusions

Mercury intoxication presents many different symptoms, depending on the form and the route of entry to the organism. Inhalation of metallic or inorganic mercury vapour mainly induces pulmonary disease while chronic inhalation induces neurological or renal disorders [5,6,12]. There have been rare cases of acute renal failure, acute respiratory distress, hemorrhagic colitis secondary to inhalation, abdominal discomfort and acute chemical pneumonitis [2,5,6,16]. Nevertheless, ingestion of elemental mercury rarely causes symptoms of intoxication because it is not well absorbed in the gastrointestinal tract [16].

Pulmonic embolism, development of pulmonary empyema, and embolisation to the heart has been reported following intravenous injection [4,15-17]. Davey and Benson [15] and Alhamad et al. [5] reported metal dense deposits in the heart following intravenous injection of metallic mercury or inhalation of vaporized mercury. Intra-arterial injection can cause ischemia and/or gangrene secondary to embolisation [10].

In general, there are no clinical signs of acute systemic intoxication following subcutaneous and intramuscular injection of elemental mercury. An intense local inflammatory reaction with formation of granulomas or aseptic abscess formations may occur several weeks or months following injection [4,5,7,9,10,14-17]. Late reactions include foreign body giant cell reaction, fibrosis, granulomas and membranous fat necrosis [4,10,16]. Schwarz et al. [18] reported a case of amyotrophic lateral sclerosis 3 years after injuring the hand with a clinical thermometer and Benz et al. [19] described white matter lesions in a 4-year-old girl with chronic intoxication of inorganic mercury secondary to the accidental use of a skin whitening cream.

Nevertheless, following subcutaneous injection of metallic mercury it is essential to prevent systemic absorption and to treat local effects properly. Therefore, prompt marginal or wide excision of subcutaneous deposits has to be done according to Enneking et al. [4,7,9-11,14-17]. Furthermore, monitoring of the central nervous system and renal functions should be performed. Chelating therapy and psychiatric consultation should be applied when indicated [4,9-11,14,16].

Ellabban et al. [9] reported that subcutaneous deposition of mercury does not carry the systemic risk associated with mercury inhalation. Chodorowski et al. [20] reported two deaths secondary to renal failure and pulmonary empyema [10]. On the other hand, Davey and Benson [15] suggest that the chronic phase of metallic mercury intoxication following intravenous injection seems to be benign, with low risk of developing long term neurological or pulmonary sequel.

In the present case, there have been no symptoms of mercury intoxication during a period of nearly 15 years of exposure. However, prolonged clinical and biochemical follow-up have to be performed [4,11,17], although it is known that there is no correlation between mercury levels in the blood and urine and the severity of symptoms in case of chronic poisoning [7,12,21].

The usage of chelating agents (e.g. DMPS, dimercaprol (BAL), dimercaptosuccinic acid (DMSA), British Anti Lewisite (BAL), d-penicillamine) in the treatment of mercury intoxication is controversial [4-6,8,15,16]. Sarikaya et al. related the usage of N-acetyl cysteine (NAC) for chelating therapy, additionally. Several authors recommended the usage of chelation therapy if there are signs of systemic intoxication and following intravenous injection [3,5,7,9-11,14,21]. On the other hand, Davey and Benson [15] suggest that the long term benefit of chelation therapy is equally low in case of chronic mercury poisoning and therefore the usage should be restricted to acute intoxication, while Lim et al. [6] reported no effect on progression of acute lung injury.

In the present case, chelating therapy with DMPS was introduced due to the recommendation by the local centre for intoxications, although there were no signs of systemical poisoning. Furthermore, haemodialysis and plasma exchange are only indicated if renal failure occurs combined with anuria [5,13]. Nevertheless, Yoshida et al. [13] suggested that renal damages due to acute mercury poisoning may not be permanent.

Summarizing, blood levels of mercury will decrease rapidly following surgery combined with chelating therapy. Subcutaneous and intramuscular injection of metallic mercury requires marginal or wide excision of all contaminated tissue to prevent migration of mercury and chronic inflammation or intoxication. Nevertheless, prolonged clinical and biochemical monitoring should be performed for several years to screen for chronic intoxication.

## Competing interests

The authors declare that they have no competing interests.

## Authors' contributions

JF, WME, and PS acquired the data and wrote/revised the manuscript, ET performed the clinical follow-up of the patient, AL and WME performed the surgery. Furthermore, all authors have been involved in drafting the manuscript or revising it critically for important intellectual content read and approved the final manuscript.

## Pre-publication history

The pre-publication history for this paper can be accessed here:

http://www.biomedcentral.com/1471-2482/11/31/prepub
